# Tumor-Infiltrating Lymphocytes and Their Prognostic Value in Cutaneous Melanoma

**DOI:** 10.3389/fimmu.2020.02105

**Published:** 2020-09-10

**Authors:** Fabienne Maibach, Hassan Sadozai, S. Morteza Seyed Jafari, Robert E. Hunger, Mirjam Schenk

**Affiliations:** ^1^Institute of Pathology, Experimental Pathology, University of Bern, Bern, Switzerland; ^2^Department of Dermatology, Inselspital, Bern University Hospital, Bern, Switzerland

**Keywords:** melanoma, tumor infiltrating lymphocyte, prognostic marker, tumor immunology and microenvironment, immunotherapy, tertiary lymphoid structure

## Abstract

Recent breakthroughs in tumor immunotherapy such as immune checkpoint blockade (ICB) antibodies, have demonstrated the capacity of the immune system to fight cancer in a number of malignancies such as melanoma and lung cancer. The numbers, localization and phenotypes of tumor-infiltrating lymphocytes (TIL) are not only predictive of response to immunotherapy but also key modulators of disease progression. In this review, we focus on TIL profiling in cutaneous melanoma using histopathological approaches and highlight the observed prognostic value of the primary TIL subsets. The quantification of TIL in formalin-fixed tumor samples ranges from visual scoring of lymphocytic infiltrates in H&E to multiplex immunohistochemistry and immunofluorescence followed by enumeration using image analysis software. Nevertheless, TIL enumeration in the current literature primarily relies upon single marker immunohistochemistry analyses of major lymphocyte subsets such as conventional T cells (CD3, CD4, CD8), regulatory T cells (FOXP3) and B cells (CD20). We review key studies in the literature on associations between TIL subsets and patient survival. We also cover recent findings with respect to the existence of ectopic lymphoid aggregates found in the TME which are termed tertiary lymphoid structures (TLS) and are generally a positive prognostic feature. In addition to their prognostic significance, the existence of various TIL sub-populations has also been reported to predict a patient’s response to ICB. Thus, the literature on the predictive potential of TIL subsets in melanoma patients receiving ICB has also been discussed. Finally, we describe recently developed state-of-the-art profiling approaches for tumor infiltrating immune cells such as digital pathology scoring algorithms (e.g., Immunoscore) and multiplex proteomics-based immunophenotyping platforms (e.g., imaging mass cytometry). Translating these novel technologies have the potential to revolutionize tumor immunopathology leading to altering our current understanding of cancer immunology and dramatically improving outcomes for patients.

## Introduction

Over the past two decades, tumor immunotherapy has demonstrated remarkable clinical success for a number of different types of cancers (1, 2). While a large variety of drugs are currently in clinical trials for tumor immunotherapy, the overall therapeutic aims of these agents are to disrupt or counteract tumor-mediated immunosuppression (3, 4). The most clinically effective immunotherapies to date are monoclonal antibodies targeting the checkpoint molecules CTLA-4 (cytotoxic T lymphocyte-associated protein 4) and PD-1 (programmed cell death protein 1) as well as its ligand PD-L1 (4–6). While there is significant variability in the response rates to these drugs based on the tumor type, a recent pooled analysis demonstrated an average objective response rate (ORR) of 14% for anti-CTLA-4 and 33% for anti-PD-1 mAbs (7). In a separate meta-analysis of results from phase III trials with ICB, it was shown that patients treated with ICB were over 2 times more likely to experience durable clinical responses compared to patients in the control (non-ICB) arms of these trials (8). Thus, tumor immunotherapy is established as a key therapeutic approach for the clinical treatment of cancer.

The successful response to immunotherapies is predicated on the immunological composition or “immune contexture” of the tumor microenvironment (TME) (9–11). Furthermore, the increased presence of certain cell types has also been shown to be associated with enhanced survival in patients with various forms of cancer (10, 12). These observations are in accordance with the canonical understanding that a healthy immune system regularly eliminates pro-tumorigenic cells and can mount responses to established cancers which eventually escape immune control and progress, a concept currently known as “cancer immunoediting” (13, 14). The processes through which the immune system both restricts and promotes tumorigenesis remain a key focus of current immunology research (14, 15). As such, an assessment of tumor infiltrating immune cells, in particular tumor infiltrating lymphocytes (TIL) is of critical importance in biomedical research as well as clinical pathology (10, 12, 16).

Cutaneous melanoma is the most lethal form of skin cancer (17). The incidence of cutaneous melanoma continues to rise in Europe and 5-year survival rates remain low (<30%) despite the advent of tumor immunotherapies (18). In both preclinical and clinical studies on melanoma, examination of TIL and various TIL subsets by histopathologists has yielded several biological insights in tumor immunology (10, 19). For over three decades, a number of clinical centers have demonstrated the therapeutic potential of adoptive TIL transfer in melanoma (2). However, the technical challenges and costs associated with this method have limited a wider application of this approach and the development of transgenic T cell receptor bearing T cells remains an important field of immunotherapy research. Nevertheless, as the mechanistic and prognostic relevance of various TIL subsets in cancer remains a key avenue of research, it is imperative to understand current approaches to TIL profiling and their association with patient clinical outcomes (12). While techniques, such as multi-color flow cytometry and mass cytometry (CyTOF) currently permit high dimensional immunophenotyping of human tissues, these procedures require tissue dissociation which leads to loss of crucial spatial and morphological information (20). Thus, clinical pathology on fixed tissue sections remains the gold standard for examining the TME *in situ*. Generally, routine histopathology is performed on whole tissue sections but in recent years, a large number of investigational studies have started to use tissue microarrays (TMA) for biomarker discovery and immunological or molecular profiling in cancer research (12, 21).

In this review, we explore the prognostic relevance of TIL pattern profiling enumerating various lymphocyte subsets. We also examine their predictive value for ICB treatment. Moreover, novel algorithms for histological assessment of tumors such as the ImmunoScore method as well as machine-learning based image analysis will be discussed. Finally, novel methodologies for high-throughput immunophenotyping of tumors will be described. Collectively, this work provides a scientific primer on TIL subsets in melanoma and highlights novel immunophenotyping techniques which harbor significant promise for advancing our understanding of tumor-infiltrating lymphocytes.

## Profiling of TIL in Cutaneous Melanoma

### TIL Assessment in H&E

Following the first characterization of TIL in melanoma by Clark in 1969, multiple groups reported that lymphocyte infiltration correlated with improved survival in melanoma patients (22). While research has shown that immune cell subsets are highly heterogeneous within human cancers, enumerating TIL using H&E provides an important insight on the TME in a number of cancer types (12, 23). Histologically, the location of the immune infiltrate may be described as intratumoral, stromal or peritumoral (12, 24). Intratumoral immune cells are located directly within the malignant “nest” of tumor cells (12). The stromal region is composed of blood vessels, connective tissue and various immune cells (24). The outer border of the tumor is known as the invasive front (24). The term peritumoral may be thus applied to the cells around the invasive front, in the stroma or in adjacent non-involved tissue (12, 24). Currently, there is no single unified approach for assessing overall immune infiltrates in H&E stained tissue in solid tumors. Both intratumoral and peritumoral lymphocytes may be assessed and analyzed for correlation to various clinical parameters (12). However, it is relevant to note that a vast majority of pathological analyses are currently performed using digitized whole slide images (25, 26). This allows for high resolution images, dynamic zooming and panning capabilities and permits the images to be analyzed using image analysis softwares (25). Generally, in cutaneous melanoma, TIL scoring is performed for round inflammatory cells excluding polymorphonuclear cells in the intratumoral region only (27). The biological and clinical significance of examining intratumoral versus peritumoral TIL has been discussed elsewhere (12, 28, 29). Furthermore, while a majority of studies examining TIL have been performed using primary melanoma tumors, some groups have also studied the prognostic value of examining TIL in metastatic samples (30–32).

**FIGURE 1:**
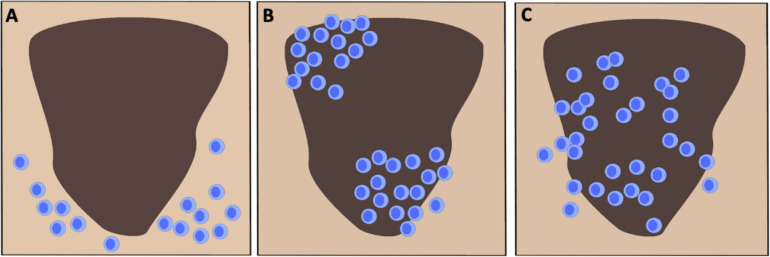
Schematic representation of the three canonical TIL infiltration patterns in cutaneous melanoma. **(A)** Absent: No presence of lymphocytes in the tumor or no infiltration within the tumor itself. **(B)** Non-brisk: One or multiple scattered foci of lymphocytes. **(C)** Brisk: Diffuse infiltration of lymphocytes throughout the tumorigenic vertical growth phase or along the tumor base.

There are presently two primary strategies for scoring TIL in H&E stained melanoma tissue, the system devised by Clark and colleagues and the approach promoted by the Melanoma Institute Australia (MIA) (12, 33, 34). The Clark scoring system was first reported in 1989 and defines three distinct TIL patterns as absent, non-brisk and brisk (Figure 1) (23, 35). Absent indicates when no TIL are present or they do not infiltrate the tumor (27, 35). Non-brisk denotes one or more scattered foci of lymphocytes (22, 35). Brisk describes a diffuse infiltration of lymphocytes throughout the tumorigenic vertical growth phase (VGP) or along the base of the tumor (23, 35, 36). Clemente et al. further divided the scoring of brisk TIL patterns into peripheral (along the tumor base) or diffuse (infiltrating the entire invasive portion of the tumor) (23, 37). Generally, perivascular lymphocytes and lymphocytes in regions of fibrosis are not included in the scoring (23). The Clark system remains in wide usage due to its reproducibility, ease of application and strong interobserver agreement (23, 38). The MIA scoring system is based on the density (mild, moderate or marked) and distribution of TIL (focal, multifocal, or diffuse across the entire tumor) in the dermis (23, 34). The MIA ordinal score (0–3) is defined as follows: grade 0; TIL absent, grade 1; a mild multifocal or a mild/moderate focal infiltrate, grade 2; a moderate or marked multifocal, a marked focal or a mild diffuse TIL pattern, grade 3; a moderate or marked diffuse infiltrate (34).

**FIGURE 2:**
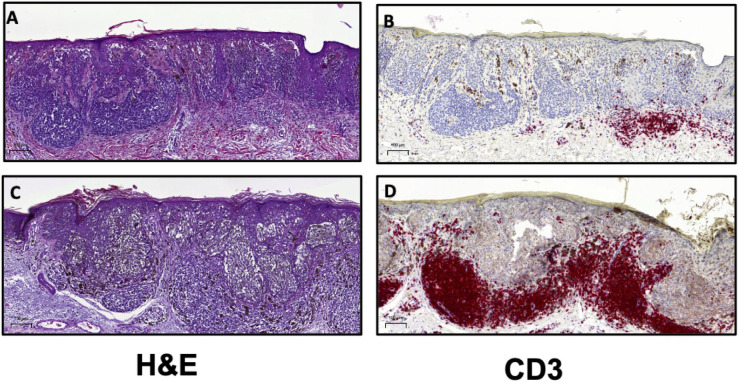
H&E and CD3 immunostaining can be utilized to distinguish T cell-inflamed and non-inflamed tumor microenvironments. Representative images from malignant melanoma samples comparing **(A)** a tumor with mild, focal lymphocytic infiltration with **(B)** few T cells compared to **(C)** a tumor exhibiting dense TIL patterns and **(D)** a high frequency of T cells. Scale bar = 100 μm.

Both the Clark and MIA scoring systems have demonstrated that increased TIL levels are associated with improved prognosis. In the aforementioned report by Clark et al., the authors studied 8-year survival in over 200 patients with exhibiting distinct histologic subtypes of primary cutaneous melanoma (35). The 8-year survival rate in patients with absent TIL was 59, 75% in patients with non-brisk TIL patterns and 88% in patients with brisk TIL (35). As a further demonstration of the biological relevance of TIL scoring, the absence of TIL as defined by Clark, was associated with increased sentinel lymph node (SLN) metastases (39). SLN status remains the most important independent prognostic factor in melanoma (40). In 2012, Azimi et al. utilized the MIA scoring system to show that TIL grade in the primary cutaneous melanoma was inversely associated with SLN positivity and was independently associated with disease-specific survival (DSS) (34). Thus, there is ample evidence to demonstrate that the presence of TIL is associated with an enhanced host response to the tumor and therefore is associated with improved patient outcomes. A recently reported meta-analysis of 41 published studies on TIL in melanoma showed that simply the presence of TIL was significantly associated with improved overall survival (OS) (33). A majority of the studies included in this meta-analysis were performed on primary cutaneous melanoma (33). The authors of this report demonstrated that brisk TIL patterns were associated with improved prognosis with respect to OS, recurrence free survival (RFS) and DSS (33). Together, these findings indicate that the presence of lymphocytes in the tumor mass is representative of a host immune response to the cancer and thus it is generally associated with positive clinical outcomes. Given the utility of H&E assessment of TIL, the International Immuno-Oncology Biomarker Working Group (IOBWG) has proposed a more standardized approach for TIL assessment (41). However, this approach requires validation in melanoma and other solid tumors in order to determine its viability for prognosis. Despite the effective use of TIL scoring as a prognostic tool in melanoma, the immune cells infiltrating a tumor are phenotypically and functionally diverse (42). The TME for most cancers is highly heterogeneous and through histological assessment, three major categories of immune infiltration have been identified (43, 44). Broadly, tumors can be classified as those with T cell-inflamed (hot) and non-T cell-inflamed (cold) TME (Figure 2) (44). Profiling of these subtypes has shown that T cell-inflamed tumors contain high density of CD8^+^ TIL, expression of PD-L1, increased IFNγ signaling and a high mutational burden (43, 44). Non-T cell-inflamed tumors generally have an immune-excluded TME with a peripheral accumulation of T cells which are unable to enter the tumor mass as a result of immunosuppressive myeloid cells or stroma (43). Alternately, cold tumors may also represent an immune-ignored phenotype with minimal or absent T cell infiltration with highly proliferative tumor cells, lack of PD-L1 expression and a low mutational burden (43, 44). The classifications described here are general categories derived from histopathological analyses of tumor tissue (43). The roles of stromal cells, mutational burden and tumor-infiltrating immune cells in driving the phenotype of the TME are still not well described and require further elucidation (43, 45). As such, IHC for the detection and enumeration of major lymphocyte subsets in the tumor and determining their correlation to patient survival or response to treatment, is also a crucial approach for clinical and pre-clinical cancer research (10, 45).

### Assessment of TIL Subsets via IHC

Tumor infiltrating immune cells are heterogeneous in both phenotype and function and form an interactive network with other immune cells and non-immune components of the TME (10, 42). Due to advances in monoclonal antibody production, it is presently possible to detect numerous leukocyte subsets in histopathology tissue samples using both IHC and immunofluorescence (IF) (10, 20, 46). However, a majority of studies use antibodies targeted to key surface markers to broadly identify major TIL subsets in cancer such as CD20 (B cells), CD3 (T cells), CD4 & CD8 (T cells) and FOXP3 (Regulatory T cells or T_reg_) (12, 33, 47). While tumor infiltrating CD8^+^ T cells have been shown to be associated with positive outcomes and FOXP3^+^ T_reg_ are usually associated with negative outcomes, there is tremendous heterogeneity in the results of IHC-based detection and enumeration of TIL subsets in cancer (10, 12, 30). However, there is no single approach for quantifying immune cells in tumor tissue. Thus, certain studies perform semi-quantitative (e.g., low/high or 0–3) grading by visual examination (48, 49), or using image analysis software to determine percentage of pixels positive for IHC staining (50). The Clark grading system of absent, non-brisk and brisk for TIL in H&E tissue has also been applied to tissue with stains for lymphocyte markers such as CD8 (51). On the other hand, the total cells positive for a specific marker (e.g., CD3) are enumerated using visual assessment or digital image analysis software (31, 52, 53). Given the variety of techniques utilized to score TIL and TIL subsets in cancer between studies, conflicting results regarding their prognostic relevance in cancer are to be expected. However, recent advances in machine learning image analysis algorithms are promising in their capacity to unify and automate digital pathology, thereby yielding more consistent results (54, 55).

In the context of melanoma, studies have shown that while the presence of intratumoral lymphocytes is generally a positive prognostic factor, inspecting individual populations using markers such as CD3 or CD8, or activated T cells, may yield conflicting results in smaller cohorts of patients (30, 33). In a recent review, Fridman et al. combined data from 200 previously published studies in various cancer types to obtain a comprehensive view of the prognostic value of major immune cells subsets (10). A key limitation in the case of melanoma versus other tumors such as colorectal cancer and breast cancer is the relative scarcity of studies examining lymphocyte subsets and their association with prognosis (10). Nevertheless, it has been observed that while T cells (CD3^+^, CD8^+^, CD4^+^) and B cells (CD20^+^) are associated with better patient outcomes, FOXP3^+^ T_reg_ are associated with worse prognosis (Table 1) (10, 12, 30, 56). In this section, we will review the known roles of major TIL subsets in tumor immunity as well as novel methods which might improve and refine immunophenotyping of these subsets in cancer. Finally, it is important to note that while TIL immunophenotyping primarily relies upon detection of immune cell specific protein markers, a number of studies have also performed gene expression profiling of tumor tissue to detect immune cell specific transcripts (57). These approaches can currently be complemented by examining immune cell specific transcripts in bulk tumor RNA sequencing datasets which are publicly available at The Cancer Genome Atlas (TCGA) (58). In addition to assessing individual genes, a number of “immune cell deconvolution” algorithms have attempted to estimate the relative abundance of tumor-infiltrating leukocytes based on unique gene signatures for each cell type (59). Furthermore, high or low expression of these signatures may be compared with survival data which is available in TCGA, to predict the prognostic value of various leukocyte subsets (60). While gene expression signatures can provide evidence of a tumor’s immune profile, ultimately the presence of these cells in the TME must be validated through flow cytometry or histopathology.

**TABLE 1:** Overview of TIL subset detection via IHC, their canonical functions in the tumor microenvironment and association with patient survival.

TIL subset	Primary identification marker^†^	Canonical anti-tumor function(s)	Association with patient survival^‡^	References
Cytotoxic T lymphocyte	CD8	Direct cytotoxicity to tumor cells. Production of IFNy	Positive	(31, 32, 67, 68)
			No association	(51)
Helper T lymphocyte	CD4	Production of pro-inflammatory cytokines. Direct cytotoxicity to tumor cells.	Positive	(32, 68)
			No association	(31)
Regulatory T lymphocyte	FOXP3	Direct and indirect suppression of effector lymphocytes. Inhibit APC functions.	No association	(31, 32, 93)
B lymphocyte	CD20	Production of anti-tumor antibodies. Antigen presentation.	Positive association	(31, 102)
			No association	(168)
			Negative association	(104)

*^†^Accepted as primary markers for the following cell types in the scientific literature. ^‡^Only statistically significant associations between density of each TIL subset and patient outcomes (OS, RFS, DSS, PFS) were included.*

## CD8^+^ TIL

CD8^+^ T cells play a central role in the adaptive immune response to cancer (61–63). Activated CD8^+^ T cells are termed cytotoxic T lymphocytes (CTL) as they are capable of directly recognizing and killing malignant and infected cells (61, 63). While multiple immune cells can exhibit tumoricidal activity including natural killer (NK) cells and macrophages, CTL are the primary immune cells capable of controlling tumor growth and mediating responses to cancer immunotherapies (62–64). The primary anti-tumor activities of CTL involves their killing of target cells via the exocytosis of cytotoxic granules containing perforin and granzymes as well as the production of cytokines such as IFNγ and tumor necrosis factor (TNF) (62, 63). Recent evidence shows that a subset of conventional dendritic cells (cDC), cDC1 are essential for the recruitment and priming of CD8^+^ T cells in the TME (65). As described in the aforementioned conceptual framework of cancer immunoediting, after a period of initial control by T cells, the tumor is able to evade the immune attack and promote an immunosuppressive TME (14, 61). On the other hand, chronic antigen exposure such as in the context of chronic viremia and cancer, leads to a state termed T cell exhaustion marked by loss of effector functions, a sustained expression of inhibitory surface receptors (e.g., PD-1) and distinct transcriptional profiles (66). Therefore, the diverse phenotypes and functional profiles of CD8^+^ TIL in the TME complicate the interpretation of histological studies on these cells in tumor tissues (12, 30).

In an early report CD8^+^ TIL levels in primary cutaneous melanoma tumors were found to be associated with increased survival in a cohort of 47 patients (67). By stratifying the patients based on CD8^+^ T cell density, the 5-year OS for the high, moderate and low-density groups was observed to be 78, 44, and 25%, respectively (67). However, in a larger cohort of over 180 primary cutaneous melanoma samples in 2011, they found no correlation between CD8^+^ TIL and patient survival (51). Thus, these studies demonstrate the complexity of determining the prognostic relevance of CD8^+^ TIL using immunohistochemistry. In addition to primary tumors, a number of studies have also examined the TIL profiles of metastatic lesions. These reports have shown that an increased density of CD8^+^ T cells in metastatic melanoma lesions (Stage III and IV) is positively associated with survival (31, 32). As previously discussed, CD8^+^ TIL secrete a number of functional molecules and express a wide variety of surface markers (62). Therefore, some studies have investigated the use of labeling activation markers or effector molecules in addition to labeling CD8 when studying the prognostic significance of CD8^+^ TIL. In a cohort of primary melanoma tumors (Stage II), it was shown that the presence of TIL positive for the CD8^+^ T cell effector molecule Granzyme B (GZMB) were associated with longer progression-free survival (PFS) and OS (68). Dual IF labeling confirmed that GZMB^+^ TIL were also positive for CD8 (68). Further research on the functional status of T cells in the TME has revealed a number of biologically relevant surface markers which may assist in examining the prognostic value of CD8^+^ TIL in melanoma. Flow cytometric analyses of intratumoral CD8^+^ T cells in metastatic lymph nodes (mLN) have shown that the expression of the chemokine receptors CXCR3 and CCR9, as well as the c-type lectin, NKG2D, on CD8^+^ TIL correlates with improved clinical outcomes in cutaneous melanoma (69). An important distinction in the TME is that between lymphocytes which are tumor-specific (i.e., recognize tumor antigens) and bystander TIL (70). Using a multiplexed tetramer and mass cytometry based approach, surface expressed ectonucleotidase CD39 was identified as a marker to distinguish tumor-specific CD8^+^ TIL in lung and colorectal cancers (70). In this report, the authors found that bystander TIL express inhibitory receptors such as PD-1 and TIGIT (T cell immunoreceptor with Ig and ITIM domains) which were previously reported to distinguish tumor-specific “exhausted” TIL (70, 71). While advanced immunophenotyping technologies have demonstrated the diversity of CD8^+^ TIL in the TME, the prognostic utility of labeling additional markers on CD8^+^ TIL such as TIGIT, CD39 or NKG2D in clinical and research pathology has yet to be determined. Nevertheless, assessment of CD8^+^ T cells in the TME remains one of the key readouts for intratumoral immune activation when assessing disease prognosis or determining successful response to immunotherapies (12, 72).

## CD4^+^ TIL

Similar to CD8^+^ T cells, CD4^+^ T helper (T_H_) cells exhibit tremendous diversity in phenotype and function (73). Moreover, CD4^+^ T cells are essential for cancer immunity via mechanisms that have been recently reviewed (73, 74). CD4^+^ T cells can directly kill tumor cells via cytolytic mechanisms or produce cytokines such as IFNγ which promote anti-tumor immune responses (61, 73). Furthermore, in secondary lymphoid organs CD4^+^ T cells can modulate both B cell and CD8^+^ CTL responses (74). Studies using murine models have shown that CD4^+^ T cells can enhance the potency of CD8^+^ T responses (74). CD4^+^ T cells have also been described in terms of their differentiation into various T helper lineages marked by distinct transcription factors and cytokine production (73, 75). The most studied of these lineages are T_H_1, T_H_2, T_H_17, T follicular helper (T_FH_) and CD4^+^FOXP3^+^ T_reg_ (73). While the complex functions of each subset in various tumor types remain poorly described and require further investigation, it is generally accepted that Th1 cells promote effective anti-tumor immune responses via the production of large amounts of IFNγ, promoting not only CD8^+^ CTL function but also recruiting NK cells and classically activated M1 macrophages (73). Alternately, T_reg_ are hypothesized to assist tumor growth due to their capacity to inhibit effector T cells and to mediate immunosuppression (10, 76).

As mentioned earlier, a majority of studies on the prognostic value of TIL in melanoma have utilized TIL pattern scoring in H&E tissue with very few reports examining TIL subsets *in situ* using IHC (10, 12, 30). In aforementioned study using primary cutaneous melanomas (Stage II), the authors demonstrated that while the presence of GZMB^+^ TIL was associated with longer OS and progression-free survival (PFS), the presence of CD4^+^ and CD8^+^ TIL was associated only with improved PFS and not OS (68). However, in the context of metastatic melanoma, there are no conclusive studies demonstrating the prognostic significance of CD4^+^ TIL assessment using histopathology (12, 30). A recent report using multi-parameter flow cytometric profiling revealed that proportions of naive CD45RA^+^CD4^+^ T cells in mLN of stage III cutaneous melanoma patients, inversely correlated with the frequencies of CD8^+^ T cells (69). Furthermore it was observed that patients with markedly higher proportions of naive CD45RA^+^CD4^+^ T cells in their tumors exhibited significantly reduced PFS (69). Finally, the surface markers CD69 and PD-1 were also found to be expressed on CD4^+^ T cells in metastatic tumors but the prognostic value of assessing these markers using immunohistochemistry or *in situ* IF remains to be demonstrated in melanoma (69).

Currently, only a limited number of studies have investigated the prognostic potential of CD4^+^ TIL enumeration in melanoma using IHC or IF (12, 30). Using TMAs constructed from metastatic melanoma samples (from multiple anatomic sites) and IHC to identify major TIL subsets, it was shown that while higher densities of CD3^+^ and CD8^+^ TIL were positively associated with OS, this was not the case for CD4^+^ TIL (31). Nevertheless, a study which examined only melanoma metastases within the SLN and enumerated intratumoral lymphocytes by visual counting, higher counts of CD4^+^ TIL were significantly correlated to increased OS and RFS (32). As SLN biopsy is routinely performed to stage primary cutaneous melanoma, assessment of various TIL subsets within metastatic SLN may provide useful prognostic and biological insights on the roles of these cells in cancer immunity (77). However, the studies mentioned above complicate the interpretation of the roles of CD4^+^ TIL in melanoma. First, the low number of studies examining TIL subsets in melanoma and the diverse techniques used to identify and enumerate labeled cells do not allow for standardized comparisons between multiple reports (12, 30). Second, it is not possible to characterize the diversity of CD4^+^ T helper lymphocytes by labeling only the surface antigen CD4. While T_H_1 CD4^+^ TIL are considered to augment cancer immunity, the roles of T_H_2 and T_H_17 are more nuanced and their involvement in tumor development and progression are not fully understood (73, 78). Knowledge of the mechanisms through which T helper subsets influence tumor development has been largely obtained from *in vivo* murine models where both CD4^+^ T_H_1 and T_H_2 cells have been shown to eliminate B16 melanomas (78). However, studying T helper subpopulations is challenging in the context of immunopathology as they often do not express unique surface markers and are defined by the differential expression of key cytokines (73). As a result, a number of studies have performed gene expression profiling to assess T_H_1 or T_H_2 signature genes in human melanoma biopsies. A report demonstrated that the expression of T_H_1 associated genes such as TNFβ and IL-2 was significantly higher in primary melanoma tumors which undergo spontaneous regression (a clinically observed occurrence indicating the activation of host anti-tumor immune responses) compared to non-regressing tumors (57). Gene expression of the primary T_H_1 effector cytokine, IFNγ, was also elevated in regressing primary tumors but these levels did not reach significance (57). In another study, CD4^+^ T cells from SLN of 13 cutaneous melanoma patients (positive and negative LN were included) displayed a T_H_2 skewed gene signature in association with increased production of VEGFA (79). While the differentiation of T cells into various effector subpopulations is still not fully understood, it is known that specific transcription factors regulate the differentiation of CD4^+^ T cells into various T helper lineages such as T_H_1 (T-bet), T_H_2 (GATA-3) T_H_17 (RORγt) and T_reg_ (FOXP3). Thus, in some settings, IHC for T-bet^+^ cells in tumor tissue has been performed as a readout for T_H_1 cells. In primary tumors of colorectal as well as ovarian cancer, increased numbers of T-bet^+^ cells were found to be associated with improved prognosis in patients (80, 81). However, similar studies have not been reported in the literature for melanoma (12, 30, 33). Furthermore, using T-bet as a marker for T_H_1 cells in single or dual marker IHC may lead to biologically invalid assumptions as shown by recent work demonstrating that T_reg_ can also express T-bet (82). In a landmark paper, Levine et al. showed in murine models that T-bet is stably expressed by T_reg_ cells and the depletion of T-bet^+^ T_reg_ in mice resulted in pronounced T_H_1 autoimmune responses (82). Thus, the latest studies have challenged the accepted paradigms of T helper cell differentiation and as such, assessing CD4^+^ TIL in cancer tissue will necessitate the evaluation of a number of specific phenotypic markers in addition to CD4^+^ to study the biological and prognostic relevance of each subset. This is especially valid in view of the observed diversity in T_reg_ populations in cancer and the divergent results from studies which have utilized FOXP3^+^ to examine tumor-infiltrating T_reg_ (10, 83).

## FOXP3^+^ TIL (T_reg_)

Regulatory T cells i.e., T_reg_, are essential for maintaining self-tolerance and as such, are crucial for the proper functioning of a healthy immune system (76, 84). However, in the context of cancer, T_reg_ can limit anti-tumor immune responses thereby contributing to an immunosuppressive TME (76). To date, the best characterized population of regulatory T cells are CD4^+^CD25^+^FOXP3^+^ T_reg_ and in the literature, the term T_reg_ is primarily applied to that aforementioned subset (84). CD4^+^ regulatory T cell populations which lack FOXP3 expression such as Tr1 (Type 1 regulatory) have also been described but their specific functions are yet to be fully characterized (84). Moreover, the expression of FOXP3 is not restricted to CD4^+^ T_reg_ and has been reported in both normal and neoplastic epithelial tissue as well as in other immune cells such as CD8^+^ T cells (76, 85). Similar to CD4^+^FOXP3^+^ T_reg_, CD8^+^FOXP3^+^ T cells with apparent immunosuppressive capacity have been reported in human malignancies such as colorectal cancer and ovarian cancer (76). Nonetheless, CD4^+^CD25^+^FOXP3^+^ T cells remains the central focus of T_reg_-based research in cancer biology due to their potent immunosuppressive functions and their presence in a wide range of tumor types (76, 86). The recently observed diversity within T_reg_ populations and the mechanisms through which T_reg_ suppress immune responses have been reviewed elsewhere (76, 86, 87). The major mechanisms of T_reg_ immunomodulatory functions can be divided into four categories as follows: a) release of immunosuppressive cytokines such as IL-10, IL-35 and TGFβ, b) direct cytotoxicity to activated cells via granzymes and perforin, c) direct regulation of APC function through surface expression of the checkpoint molecule CTLA-4 and d) nutrient deprivation of effector cells through high surface expression of the IL-2 receptor subunit α (CD25) leading to reduced IL-2 levels for effector T cell activation (87–89). Canonically, T_reg_ were also purported to induce metabolic disruption of activated immune cells through the surface molecules CD39 and CD73 which converts ATP to AMP (via CD39) and eventually to adenosine (via CD73) leading to immunosuppressive signaling in effector T cells and APC through the adenosine A_2__A_ receptor (A_2__A_R) (86, 87). Nevertheless, a recent study suggests that in human cancers, CD73 is expressed on only a limited proportion of T_reg_ and a higher proportion of conventional T cells, while CD39 is the more commonly expressed ectonucleotidase on intratumoral T_reg_ (90). Blocking both the surface bound and soluble form of CD39 and CD73 through antibodies was recently shown to synergistically rescue human T cells from ATP-mediated suppression, suggesting the utility of such an approach for cancer immunotherapy (91). Due to the broad range of immunoregulatory capacities of T_reg_, they play an important role in promoting an immunosuppressive TME (87). Murine models of cancer have shown that depleting T_reg_ (using specific ablation of Foxp3 or anti-CD25 antibodies) lead to enhanced anti-tumor immune responses, demonstrating the biological significance of these cells to tumor progression (86). T_reg_ proportions are increased in malignant tissue, while T_reg_ make up 2–5% of all CD4^+^ T cells in the peripheral blood of healthy individuals, between 10 and 50% of CD4^+^ T cells in tumors are reported to be Treg (87). An important distinction between Treg is determined on the basis of their origin. Natural or thymic T_reg_ (tT_reg_) originate in the thymus and have key functions in the maintenance of self-tolerance in healthy individuals, while in the periphery, induced T_reg_ (iT_reg_) can be generated as a result of a number of stimuli (87). The transcription factor Helios and the transmembrane protein neuropilin 1 have been proposed to be markers for tT_reg_ but recent research has shown that these markers may not be exclusive to tT_reg_ as Helios expression has been seen in both tT_reg_ and iT_reg_ (76, 87).

In histopathology, FOXP3 remains the primary marker for detection of T_reg_ in fixed tumor tissues. FOXP3^+^ TIL are associated with poor prognosis in most cancers including breast cancer, lung cancer and pancreatic cancer, but in the case of colorectal or gastric cancer, FOXP3^+^ TIL correlate to improved clinical outcomes (10). In cutaneous melanoma, a number of reports have shown that FOXP3^+^ TIL are associated with a negative prognosis, while others have reported no significant association (10, 30). As discussed earlier for both CD4^+^ and CD8^+^ TIL, the staining approach and the enumeration method for FOXP3^+^ T_reg_ varies between studies which may account for the inconsistency between multiple reports. In a 2007 report, studying two groups of melanoma patients who either exhibited or did not exhibit local disease recurrence, it was shown that the percentage of CD4^+^ T cells which were CD25^+^FOXP3^+^ (as determined by dual IHC) was significantly elevated in the primary tumors of patients who had recurrent disease (92). This study therefore demonstrates that the methodologies used to detect, enumerate and compare FOXP3^+^ TIL are vital to determine their association with clinical parameters. In a subsequent study of primary tumor biopsies from over 90 melanoma patients, FOXP3 was detected via IHC and enumerated visually both in the intratumoral and stromal regions but failed to show a significant associations with patient survival or any other clinical parameter such as tumor thickness or ulceration (93). In a TMA consisting of metastatic melanoma lesions (multiple anatomic sites) from over 140 patients, FOXP3^+^ cell density did not show a prognostic association with survival (31). However, the functional and prognostic relevance of T_reg_ in a tumor may vary depending on whether it is a primary tumor or a metastatic lesion. In the aforementioned report by Kakavand et al. who examined metastatic SLN, a strong trend was observed for a negative association between high counts of FOXP3^+^ TIL and recurrence-free and overall survival (32). As for other immune cell genes, qPCR for FOXP3 can also be performed as a readout for T_reg_ abundance and in a study of metastatic SLN, patients with very high levels of FOXP3 gene expression (>90th percentile) displayed significantly lower PFS (94). While gene expression profiling is not routinely utilized in histopathology to study the diversity of TIL subsets, the studies discussed here show that gene expression profiling may provide a useful immunophenotyping tool in the TME. In addition to qPCR, mRNA fluorescent *in situ* hybridization (FISH) provides a useful tool for immunophenotyping tumor tissue. For instance, mRNA FISH was utilized to detect PD-L1 mRNA expression in primary breast carcinomas and was shown to be associated with longer RFS (95). In addition to CD25 and CTLA-4, T_reg_ also express a number of other key surface molecules such as ICOS, OX40, GITR and TIGIT (86, 87). However, as these markers are not exclusive to T_reg_, detection of FOXP3 in tumor tissue is inferred to represent CD4^+^CD25^+^FOXP3^+^ T_reg_ (12, 30). Nonetheless, recently work has demonstrated that a population of FOXP3^+^ non-T_reg_ cells are also found in cancer (87). As such, FOXP3^+^ populations in CD4^+^ T cells are currently classified into three fractions based on their expression of CD45RA and FOXP3. These include naive T_reg_ (CD45RA^+^FOXP3^lo^CD25^lo^CD4^+^), effector T_reg_ (eT_reg_, CD45RA^–^FOXP3^hi^CD25^hi^CD4^+^) and non-T_reg_ (CD45RA^–^FOXP3^lo^CD25^lo^CD4^+^) (87). FOXP3^+^ non-T_reg_ are considered immunostimulatory and may account for the reported association between increased FOXP3^+^ TIL and improved prognosis reported for certain cancers such as colorectal and gastric cancer (87). Therefore, FOXP3^+^ TIL represent a heterogeneous population of cells and further research is required to identify markers to clearly differentiate immunosuppressive FOXP3^+^ T_reg_ from FOXP3^+^ non-T_reg_, for their use in histopathology and therefore allow immunologists to better study classical immunosuppressive T_reg_ in tumor tissues. Furthermore, as for other TIL subsets, a standardization of methodologies for IHC detection and enumeration of T_reg_ will allow for the pooling of results between multiple studies in order to determine the biological roles of both immunostimulatory and immunoregulatory lymphocytes *in situ* not only in tumor tissues but also in autoimmune disease and organ transplantation.

## Tumor-Infiltrating B Cells

Currently, the roles of B cells in tumor immunity are not fully characterized and studies examining their prognostic potential are limited (56, 96). Cancer patients are observed to produce antibodies to a wide range of tumor antigens, in particular, overexpressed antigens or neoantigens resulting from somatic mutations (96, 97). Moreover, autoantibodies are also detectable in a wide number of malignancies (96). While antibodies specific to tumor cells can in theory, lead to their elimination through complement activation, macrophage phagocytosis and NK cells, at present there is not sufficient evidence to indicate whether autoantibodies or tumor-specific antibodies can independently control disease progression in cancer (96, 97). However, B cells also display important effector functions apart from the production of antibodies and experimental evidence has shown that B cells can both restrain and promote anti-tumor immune responses (96–98). Briefly, B cells can hinder tumor progression by promoting cytotoxicity to tumor cells, producing tumor-specific antibodies and as APC, particularly when DC function may be impaired or where DC may be absent (96, 97). Conversely, murine models have demonstrated a wide range of potentially pro-tumorigenic activity of B cells. For instance, circulating immune complexes resulting from B cell produced IgG induce chronic inflammation and myeloid cell activation while B cell secreted lymphotoxin can promote the growth of cancer cells (97). Recently, a number of studies have also shown the important role of B cell subsets with immunoregulatory function, i.e., B_reg_ (96, 99). Currently, hypothesized to be essential for maintaining self-tolerance, numerous B cell subsets display B_reg_ function defined primarily by their production of IL-10 and in certain subsets, IL-35 (99). An additional important subset of tumor associated B_reg_ were described in mice by Olkhanud et al. in 2011 (100). These CD25^+^CD19^+^B220^+^ cells termed tumor-evoked B_reg_ (tB_reg_) were observed in a murine breast cancer model (4T1) and were found to produce high levels of TGFβ (100). In a TGFβ-dependent manner, these cells were further shown to induce T_reg_ from conventional CD4^+^ T cells and were observed to be vital in promoting lung metastasis in this model (100). The authors also found evidence for the existence of such a population in humans as CD19^+^ B cells from healthy donor blood, upregulated CD25 and suppressed T cell proliferation following treatment with ovarian and colon cancer cell conditioned medium (100). The capacity to induce T_reg_ and promote metastasis suggests an essential role for this subset in disease progression. Due to their diversity of function and phenotype, B_reg_ are now recognized as a crucial immunoregulatory population in human cancers (12, 56).

A recently reported systematic review of 69 studies in 19 different types of cancers showed that most studies identified a positive or neutral association of B cells with survival, with fewer than 10% reporting a negative association (56). The most commonly used IHC marker for B-TIL is CD20, which detects both naive and memory B cells, but not differentiated plasma cells (PC), which are identified in most IHC studies as CD138 (syndecan-1) positive (12, 56). Other IHC markers utilized for PC include the immunoglobulin kappa constant (IGKC) as well as the ER-associated protein p63 (56). The transcription factor Pax5 is also used to establish the B cell lineage of lymphoid cancers (101). However, according to a recent meta-analysis, its use as an IHC marker for B-TIL in tumors has not been investigated (56). As previously described for other TIL subsets, *in situ* studies have revealed tumor-infiltrating PC and CD20^+^ B-TIL to be associated with both positive and negative prognostic outcomes (56). Relatively few studies have examined B-TIL in cutaneous melanoma with only two reports demonstrating a positive, one showing no effect and one revealing a negative prognostic association of CD20^+^ B cells (56). In 2011, Ladányi et al. showed that increased numbers of both intratumoral and peritumoral CD20^+^ B-TIL in cutaneous primary melanomas were associated with increased patient survival (102). In this report, activated T cells were also enumerated using the markers CD25 and OX40 (CD134). By combining B cell counts with activated T cell counts, patients were classified as high or low for CD20 and CD25 or OX40. It was observed that patients with high B cell and high activated T cells demonstrated the highest 5-year survival rates in the cohort while patients from the low B cells/low activated T cell subgroup displayed the poorest survival of all subgroups (102). In a more recent report in 2016, high numbers of CD20^+^ B cells in primary cutaneous melanomas with a Breslow thickness >1 mm were associated with improved survival (103). In addition, analysis of gene expression data from TCGA for over 300 cutaneous melanomas (primary tumors and metastatic tissues) showed CD20/CD19-high patients to exhibit significantly improved OS (103). In both of the aforementioned reports, the authors also found that primary tumors from patients with metastases displayed lower numbers of B cells compared to tumors from patients without metastases or in the case of the earlier report, patients without visceral metastases (102, 103). Therefore, these studies suggest that CD20^+^ B-TIL might serve an important role in promoting tumor immunity and limiting disease progression in melanoma. Further evidence for this notion is available from a study in metastatic melanoma lesions (multiple anatomic sites), where high numbers of both CD20^+^ B cells and CD138^+^ PC were found to be associated with improved survival (31). In the same report, they demonstrated that higher numbers of CD8^+^ TIL but not CD4^+^ TIL were associated with increased survival in metastatic melanomas (31). Thus, further studies are required to elucidate potential mechanisms through which B cells may promote CD8^+^ TIL responses. While it is known that B cells can produce immunostimulatory cytokines and act as APC, their precise effects on T cells in the TME remain poorly characterized (56). In contrast to the reports described above, a negative prognostic association has also been observed for B-TIL and PC in melanoma. In a study of 91 primary cutaneous melanomas where both CD3^+^ TIL and CD20^+^ B-TIL were enumerated, the latter were found to be associated with poor survival in cases where they represented 15% or more of all TIL (104). As this study utilizes a cut-off of CD20^+^ TIL as a proportion of CD3^+^ and CD20^+^ TIL, a comparison of their results to other studies which determined an association between survival and absolute numbers of B-TIL is particularly challenging. Furthermore, these findings are not in corroboration with studies in larger cohorts discussed above or with mechanistic studies in murine models. Depletion of B cells with an anti-CD20 antibody in B16 melanoma bearing mice resulted in increased tumor volume, metastatic capacity and impaired T cell immunity (105). As a whole, it can be inferred that while certain B cell subsets such as B_reg_ may be immunosuppressive (99), improved survival of patients with high CD20 gene expression and with high numbers of CD20^+^ B-TIL in the majority of patient cohorts reported thus far, strongly supports an anti-tumor role for conventional B cells in cutaneous melanoma. In a study of primary cutaneous melanomas, it was observed that cases with high levels of PC (CD138^+^) displayed worse OS than those without any PC (106). Notably, however, cases with low numbers of scattered PC displayed better OS compared to cases with high PC scores (106). These observations suggest that both the functional diversity as well as the spatial localization of B cells and PC are important considerations when determining their prognostic and biological significance in the TME. Notably, the formation of ectopic lymphoid aggregates termed tertiary lymphoid structures (TLS) has been noted in many types of tumors and these TLS are zones of B cells, T cells and DC akin to secondary lymphoid organs (SLO) (107). As such, the prognostic and mechanistic significance of the localization of B-TIL and other leukocytes within TLS is a vital avenue of investigation in tumor immunology and cancer histopathology (107–109).

## Tertiary Lymphoid Structures

TLS are transient ectopic lymphoid tissues which develop in the context of chronic inflammatory responses such as in chronic viral infections, autoimmune diseases, allograft rejection and cancer (108, 110). TLS in the tumor can vary in complexity from lymphocyte clusters to highly organized, spatially segregated structures bearing a strong resemblance to SLO, in particular, LN (107, 108). Thus, with the exception that highly organized TLS are not surrounded by fibrous capsules akin to LN, they are nevertheless recognized histologically by many of the same features as LN (107). TLS are also observed to contain high endothelial venules (HEV), distinct T cell zones with mature DC and B cell follicles with a germinal center, and evidence of antibody class-switching (107, 108). The precise mechanisms through which TLS are induced in tumors are not fully described and the research pertaining to TLS neogenesis has been recently reviewed (107, 108). Studies demonstrate that TLS generation in tumors involves many of the same molecular signals as the formation of LN, such as lymphotoxin, CCL21 and CXCL13 (108, 109). However, instead of lymphoid tissue inducer (LTi) cells which modulate LN formation, multiple cell types have been shown to be involved in the generation of TLS including DC, naive B cells, macrophages, T_H_17 and NKT cells (108). While significant further research is required to uncover the mechanisms through which TLS form and to determine their exact roles *in situ*, the induction of TLS as a therapeutic approach has been examined in murine models (107, 109). These strategies have varied from use of the LTβR ligand LIGHT (TNFSF14) to adoptive transfer of DC expressing CCL21, and have shown efficacy in a number of diverse tumor models (109). Nevertheless, the therapeutic use of TLS induction necessitates a more detailed understanding of their roles in tumor immunity.

Histologically, TLS are found in a proportion of cancers of various types such as NSCLC, melanoma and colorectal cancer (107, 109). They are usually located in the invasive margin or in the stroma rather than the tumor core, and HEV are detected in close proximity to TLS (109). Using H&E, mature TLS can be identified either as well differentiated lymphoid follicles which include structures resembling germinal centers (109). TLS can be identified by IHC using the marker DC-lysosome-associated membrane glycoprotein (DC-LAMP) for mature DC, or CD20^+^ B cell follicles next to a CD3^+^ T cell zone (109). In addition to these markers, TLS also contain CD68^+^ macrophages, CD138^+^ PC and HEV expressing peripheral node addressin (PNAd) and vascular addressin (MECA-79) (109). Finally, a number of gene expression profiles, comprised of genes for key chemokines such as CCL19, CCL21 and CXCL13, or a combined T_H_1 and B cell signature, have been utilized to determine the presence of TLS in tumor tissues (111, 112). An increasing body of evidence indicates that TLS are potent modulators of the immune response in tumors where they are found (109, 113). Generally, tumors with high TLS presence are also marked by features of CD8^+^ T cell activation, a T_H_1 tilted CD4^+^ repertoire and mature DC (108, 109). Furthermore, TLS are also purported to be involved in the generation of tumor-specific antibody responses and support antigen presentation and activation of T cells via B-TIL (109). This crucial interaction between B-TIL and T cells is further supported by the previously discussed systematic review, where the prognostic effect of CD3^+^ and/or CD8^+^ TIL was stronger when B cells (CD20^+^ B-TIL or PC) were present (56). Given these observations, it is unsurprising that increased TLS density or specific TLS features such as HEV, has been shown to be associated with improved survival in a range of different tumor types including lung cancer, colorectal cancer, pancreatic cancer and melanoma (107, 109). Nevertheless, there are a few studies which have shown that TLS may also be negatively associated with patient outcomes. In patients with hepatocellular carcinomas (HCC), a histologically validated TLS gene signature was found to be associated with poor patient survival and subsequent investigations in an HCC murine model revealed that TLS served as a niche for progenitor HCC tumor cells (114). These findings are in accordance with additional observations in the literature that TLS in a breast cancer cohort were associated with a higher tumor grade, and with murine studies showing that T_reg_ can also be found in TLS and control anti-tumor immune responses (115, 116). Thus, the mechanisms underlying whether TLS adopt immunosuppressive/pro-tumorigenic or immunogenic roles in a particular TME are not elucidated and require further study.

In cutaneous melanoma, only a limited number of studies have examined the associations of TLS with patient survival and TLS are deemed to be a favorable prognostic factor in this tumor (107, 109). In a 2007 report examining DC and T cell numbers in primary cutaneous melanomas, the authors reported that while CD1a^+^ DC were detected in the intratumoral and stromal regions, DC-LAMP^+^ (mature) DC were located in peritumoral regions in association with lymphocyte aggregates (117). An increased density of both DC correlated positively with higher numbers of activated (CD25^+^ or OX-40^+^) T cells and DC-LAMP^+^ were significantly associated with longer patient survival (117). In a subsequent 2011 study by the same group they showed an association of CD20^+^ B-TIL density with improved survival in patients with primary cutaneous melanomas (102). In the same report, the authors noted the presence of follicle-like B cell aggregates in 26% of their cohort but did not observe any association of these structures with survival (102). In 2012, it was shown that fully formed TLS could be detected in a proportion (24%) of skin metastases of patients with cutaneous melanoma, with follicular dendritic cells, B cell follicles, DC-LAMP^+^ DC and PNAd^+^ HEV, while primary melanomas contained HEV but not fully developed TLS (118). However, the biological roles of these structures in the metastatic process or disease progression remain to be deciphered (118). In another report from 2012, PNAd^+^ (detected by the MECA-79 antibody) HEV were found to be present in nearly two-thirds of all 225 primary melanoma tumors examined (119). Furthermore, HEV were observed in the vicinity of DC-LAMP^+^ mature DC and HEV density was strongly correlated to that of CD8^+^ and CD20^+^ TIL (119). Ultimately, the authors showed that high levels of HEV were correlated with low Clark invasion levels and lower Breslow thickness indicating that HEV play an important role in promoting tumor immunity in melanoma (119). Collectively, these findings support a role for TLS in mediating anti-tumor immune response.

These findings are also corroborated by transcriptomic profiling. As discussed earlier, the presence of TLS may also be determined using gene expression analysis (109). High expression of a 12-chemokine gene signature previously validated in colorectal cancer, was able to detect TLS in non-locoregional melanoma metastases (120). By comparing the tumors with the highest and lowest scores for this 12-gene signature, it was shown that tumors with low scores exhibited minimal or no lymphocyte infiltration, whereas high scoring samples displayed a notable lymphocyte infiltration with the presence of TLS both intratumorally as well as at the tumor-stroma interface (120). These TLS contained discernable lymphoid follicles with CD20^+^ B cells, CD86^+^ DC, and both CD4^+^ and CD8^+^ T cells but very few FOXP3^+^ Treg (120). Finally, the authors showed that the 12-gene expression score was also correlated to the survival of patients with melanoma metastases (120). These results are of particular interest, as the 12-chemokine score can be used to query microarray or RNA-seq data from tumors sample for which archival whole tissue is also available. Such tissue might then be analyzed using IHC and IF techniques to further dissect the architecture and the prognostic/predictive relevance of TLS. Nevertheless, in a recently published systematic review of TLS in cancer, the authors conducted an in-depth analysis of tumor RNA-seq profiles from TCGA for the presence of TLS genes and various cellular subtypes using immune deconvolution algorithms (109). It was shown that while the 12-chemokine gene signature was found in tumors with higher abundances of T cells, B cells and DC, there was significant heterogeneity among tumor types in their expression of the aforementioned signature (109). While cutaneous melanoma displayed the signature at high levels, uveal melanomas revealed a very low level of expression, which was also the case for tumors from other immune-privileged anatomical locations (e.g., glioblastoma) (109). As such, further research is required to determine the molecular events which lead to TLS neogenesis in each tumor type. Furthermore, the precise roles of various components of TLS in modulating tumor progression remain largely unknown, in particular, that of antibody-producing plasma cells (113). Finally, the prognostic roles of TLS have only been reported for a limited number of cohorts in various tumor types, and a larger, collective tissue repository is required in order to perform more meaningful histopathological analyses of TLS in melanoma as well as in other tumor types.

## TIL as Predictive Biomarkers for ICB Response

### Mechanism of Action of ICB Drugs

The immune composition of the tumor reflects the continuous interplay between the host immune response and the evasive mechanisms utilized by the cancer cells to ensure their survival (121, 122). Therefore, elevated numbers or proportions of specific tumor-infiltrating immune cells have been observed to show positive or negative prognostic associations with survival in multiple tumor types (10, 12). However, TIL profiling has also demonstrated predictive value for a subset of cancer patients treated with chemotherapy and radiotherapy (123–125). Furthermore, as mentioned above, it is established that cytotoxic chemotherapies and radiation treatment can enhance the immunogenicity of a tumor and shape the responses of intratumoral immune cells, albeit the fact that many of the mechanisms involved are not well described (126). Nevertheless, given the wide range of cancer therapies currently in clinical trials, including immune modulating agents and combination treatments, a vast amount of future work is required to accurately dissect the predictive value of various TIL subsets in response to these therapies. Tumor immunotherapies have currently emerged as a critical avenue of both preclinical and clinical research in oncology. The major types of tumor immunotherapies, their clinical efficacies and future therapeutic targets including novel checkpoint molecules (e.g., LAG-3, VISTA) have been reviewed expertly elsewhere (3, 4, 127). However, given that ICB with anti-CTLA-4 and anti-PD-1 antibodies functions by promoting T cell activation and the fact that it is currently utilized for the treatment of metastatic melanoma, an investigation of whether TIL subsets can serve as predictive markers for these treatments is of significant clinical value (128). Both CTLA-4 and PD-1 surface molecules, like the co-stimulatory molecule CD28 are members of the immunoglobulin superfamily but are bound by distinct ligands and have unique mechanisms of action (129). Thus, a brief description of their function is warranted. The expression of CTLA-4 is enhanced immediately following T cell activation (i.e., TCR stimulation) and by binding to the co-stimulatory molecules CD80 and CD86 with a higher affinity than CD28, CTLA-4 serves to limit T cell activation (129, 130). Given that APC provide co-stimulatory signaling to T cells via the interactions of CD80 and CD86 with CD28, CTLA-4 putatively functions in secondary lymphoid organs and is key for maintaining immune tolerance (130). Similarly, the PD-1 pathway also attenuates T cell activation and is an important mechanism in the prevention of autoimmunity in peripheral tissues (130). PD-1 is expressed on activated T cells, B cells and even NK cells (129). The primary ligand for PD-1, PD-L1 is expressed by both immune cells and non-immune cells while PD-L2 is putatively only expressed by APC in normal tissues (129). The expression of PD-L1 and to a lesser extent, PD-L2 is induced by pro-inflammatory cytokines such as IFNγ (130). Binding of PD-L1 or PD-L2 is purported to inhibit T cell activation via the tyrosine phosphatase SHP2, although recent studies have shown that SHP2 is dispensable for establishing T cell exhaustion *in vivo* suggesting the existence of multiple redundant signaling mechanisms downstream of PD-1 (130). While the molecular functions of CTLA-4 and PD-1 have been described, an increasing body of literature has shown that checkpoint blocking antibodies have distinct and unique functions on the immune system *in vivo* (129–131). However, in the clinic, anti-PD-1 antibodies (nivolumab & pembrolizumab) result in significantly higher ORR and lower toxicity profiles compared to anti-CTLA-4 antibodies (ipilimumab) (6, 132). It is pertinent to note that due to the very recent testing and implementation of these drugs, there is variability in the reported ORR for each age. The objective radiographic response rate was shown to be 15% for ipilimumab in patients with metastatic melanoma (6). Furthermore melanoma patients treated the anti-PD1 agents, nivolumab and pembrolizumab, demonstrate a response rate of between 35 and 40% while combination nivolumab and ipilimumab therapy in melanoma results in responses rates of approximately 60% (6). While ICB displays clinical efficacy in a number of tumor types including melanoma, only a fraction of patients display durable responses and certain forms of cancer, such as pancreatic ductal adenocarcinoma (PDAC), remain largely refractory to currently approved checkpoint inhibitors (6, 133). Thus, there is a significant need to identify tumor-specific features to identify potential biomarkers for successful response to ICB.

### Predictive Biomarkers for ICB Response

Studies have shown that a wide range of tumor and host-specific biological factors are associated with responses to checkpoint inhibitor therapies. As reviewed recently, these features include but are not limited to, tumor-specific genetic features (tumor mutational burden or particular transcriptional profiles), intratumoral expression of PD-L1, host-specific features such as gut microbiota and finally, the immune composition of the TME as well as the peripheral blood (134, 135). A number of reports have shown for instance, that elevated neutrophil to lymphocyte ratios in peripheral blood are associated with decreased survival in a number of cancer types treated with anti-CTLA-4 and anti-PD-1 ICB (135). A number of other features assessed in peripheral blood such as T cell clonality, monocytes, MDSC and T_reg_ cells are purported to be associated with response to ICB but require further investigation and validation (135). However, it is the immunological composition of the TME which remains a key focus of current research on the predictive potential of immune cells for ICB. As previously mentioned, numerous studies have investigated the roles of myeloid cells in modulating both the immune response to tumors as well as response to tumor therapies (135, 136). Thus, in keeping with the scope of this work, this section will specifically discuss the association of TIL with responses to anti-CTLA-4 and anti-PD-1 checkpoint drugs.

Due to the expression of PD-L1 by both tumor and immune cells, it has been investigated as a potential biomarker for determining response to anti-PD-1 ICB (134, 135). While the positive correlations of PD-L1 expression to patient outcomes have been reported in a number of studies, there is currently insufficient evidence to define PD-L1 as an independent biomarker for ICB response (134, 135, 137). It is important to note that IHC-based detection of PD-L1 has been approved as a biomarker for selecting pembrolizumab as a treatment in NSCLC patients (135). However, PD-L1 both on immune cells and tumor cells can be highly dynamic both in its spatial and temporal expression (135). As such, multiple reports have shown no association between PD-L1 status and ICB response while other reports have shown that patients with no PD-L1 expression display durable clinical responses to ICB (135). Therefore, collectively from the literature, it is evident that PD-L1 is not a comprehensive biomarker for response to ICB. This is particularly important given observations that even when PD-L1 is expressed, a pre-existing lymphocyte infiltrate is required for induction of anti-tumor immune responses with checkpoint inhibitors (134, 137).

To date, only a limited number of scientific reports have investigated TIL profiling in cutaneous melanoma and its potential association with response to ICB treatment in human patients (134, 138). In a study in 2011, 82 patients with unresectable stage III/IV melanomas treated with ipilimumab showed, that at baseline, only positive immunostaining for the markers IDO and FOXP3 in resected tumor biopsies, could significantly distinguish patients who obtained clinical benefit from those who did not (139). Indole-2,3-dioxygenase 1 (commonly referred to as IDO or IDO1) is a key enzyme in tryptophan catabolism and in tumors, high IDO1 levels derived from tumor cells and tolerogenic myeloid cells leads to the inhibition of effector T cells and NK cells (140). While baseline TIL scores in H&E stained biopsies were not elevated in patients who displayed clinical responses compared to those who did not, a majority of responding patients exhibited significant increases in TIL scores following treatment (139). In a more recent study which examined surgical biopsies (lymph nodes and subcutaneous/cutaneous metastases) from melanoma patients prior to treatment with ipilimumab, serial IHC for 11 leukocyte markers was performed and cell were visually enumerated to determine their association with response to therapy (141). In LN metastatic lesions, densities of CD4^+^, CD8^+^, FOXP3^+^, CD134^+^ and CD20^+^ lymphocytes as well as NKp46^+^ NK cells were found to be significantly higher in responders compared to non-responders (141). In separately evaluated subcutaneous and cutaneous metastatic lesions, responders displayed only higher densities of CD68^+^ and CD16^+^ leukocytes (141). These results suggest that the surgical site of the biopsy warrants further investigation when determining the predictive utility of TIL profiling. Furthermore, when all metastatic lesions were evaluated together, NKp46^+^, CD68^+^ and FOXP3^+^ cells were found to be the most statistically significantly different populations between responders and non-responders, in part corroborating the findings of Hamid et al. above (139, 141). Given that FOXP3 and IDO are markers for an immunosuppressive TME, their association with response to ICB treatment might be counter-intuitive (142). However, as demonstrated in a report by Spranger et al., T cell inflamed tumors do exhibit high levels of IDO, PD-L1 and FOXP3^+^ T_reg_ and murine studies suggest that these immunosuppressive mechanisms do not precede but are instead induced following the infiltration of CD8^+^ T cells into the tumor (143). Therefore, depending on the specific TME of a metastatic lesion, or a primary tumor as well as the timing of the biopsy, immunoregulatory cell markers such as FOXP3 might predict successful ICB response.

As mentioned previously, ICB with anti-PD-1 antibodies results in higher response rates than with ipilimumab (7). However, as is the case for ipilimumab, there is a crucial need for determining predictive biomarkers for anti-PD-1 drugs. In 2014, Tumeh et al. studied pre-treatment and post-treatment biopsies of metastatic melanomas (multiple anatomic sites) in a cohort of 46 patients treated with pembrolizumab (144). Using logistic regression, the authors were able to show that the density of CD8^+^ T cells at the invasive margin was the most optimal predictive marker for response to pembrolizumab (144). Other statistically significant predictors were CD8^+^ T cell density in the tumor, and the densities PD-1^+^ as well as PD-L1^+^ cells at the invasive margin and in the tumor (144). However, CD4^+^ T cell densities at baseline in the tumor or at the invasive margin were not shown to be predictive of response to pembrolizumab. Similar to the patients who responded to ipilimumab, most patients who responded to pembrolizumab also exhibited an increase in CD8^+^ TIL from baseline (144). While further studies are needed, it is evident that TIL have the potential to serve as effective biomarkers for tumor immunotherapy drugs. As discussed above for TIL profiling in prognostic studies in melanoma, IHC is limited in the number of markers which can be assessed simultaneously. A number of the findings made using IHC *in situ* are corroborated by results from multiparametric flow cytometric analyses of immune biomarkers for response to ICB. In a cohort of metastatic melanoma samples from patients treated with pembrolizumab or nivolumab, PFS and response to ICB were positively correlated with increased proportions of CD8^+^ T cells in tumor biopsies marked by high surface expression of CTLA-4 and PD-1 (145). The frequencies of CTLA-4^hi^PD-1^hi^ CD8^+^ T cells were shown to be independent of anatomic site of the biopsy or previous therapy, and treatment with anti-PD-1 antibodies resulted in an increase in the frequency of intratumoral CD8^+^ T cells with a simultaneous decrease in intratumoral CD4^+^ T cells (145). Furthermore, T_reg_ (defined as CD4^+^FOXP3^hi^CTLA-4^hi^) were not significantly associated with clinical responses and treatment with anti-PD-1 antibodies resulted in increased frequencies of CD8^+^ T cells (145). These findings are in accordance with the report by Tumeh et al. which showed that baseline numbers of CD8^+^ TIL and intratumoral PD-1^+^ cells were associated with clinical response to anti-PD-1 therapy (144). Taken together, these studies demonstrate that while CD8^+^ TIL density is a key biomarker for response to ICB, assessing surface markers such as CTLA-4 and PD-1 or functional molecules such as Granzyme B might identify subsets of CD8^+^ TIL with improved potential for predicting response to ICB.

Due to the heterogeneity between patients and tumor types, multi-parameter immunophenotyping techniques such as flow cytometry are of limited value and next-generation high-throughput technologies might be required identify novel immune biomarkers for ICB response. In a recent report, scRNAseq of metastastic lesions led to the identification of the transcription factor TCF7 as a marker for a subset of CD8^+^ T cells which are enriched in tumors of patients who respond to checkpoint therapy (anti-CTLA-4, anti-PD-1 or dual treatment) (146). In contrast, non-responding lesions displayed an enrichment of a CD8^+^ T cell subset expressing multiple genes liked to T cell exhaustion such as LAG3 and TIM3 (146). Subsequent immunofluorescent labeling of tissue sections from responding versus non-responding tumors with CD8 and TCF7 demonstrated that increased numbers of TCF7^+^CD8^+^ T cells were present in responders whereas increased TCF7^–^CD8^+^ T cells were present in non-responders (146). This study demonstrated the utility of high-throughput single-cell profiling approaches for identifying novel markers for clinically meaningful TIL subsets which can be readily applied to routine histopathology. However, the clinical validity of TCF7^+^CD8^+^ T cells as predictive biomarkers for ICB warrant confirmation in larger cohorts. Finally, studies have shown that checkpoint inhibitors can lead to the expansion of distinct TIL subsets, with anti-CTLA-4 and anti-PD-1 antibodies displaying differences effects on various lymphocyte subsets thereby leading to treatment-dependent shaping of the immune repertoire in tumors (147, 148). While these studies permit us to understand the intricate mechanisms through which checkpoint inhibitors mediate their therapeutic effects, selecting treatments and outcome prediction requires biomarkers which can be assessed in pre-treatment biopsies. As discussed in this section, collectively the literature suggests that TIL subsets such as CD8^+^ and FOXP3^+^ T cells are potentially useful biomarkers for predicting response to ICB. However, this requires validation in larger and better stratified cohorts, thereby permitting the identification of TIL subsets capable of predicting response to anti-PD-1 and anti-CTLA-4, respectively. Finally, while a single TIL population might not be predictive of response to therapy by itself, a combination of immune-specific and tumor-specific proteins might provide a more comprehensive marker panel for predicting checkpoint response. The TME is highly dynamic representing a continuous interplay of tumor cell and stromal cell derived factors with tumor-infiltrating immune cells and leukocyte derived molecules (149). The underlying driver mutations in cancers are key modulators for the expression of molecules which influence immune cell function (149). BRAF^V600E^ is a key driver mutation in melanoma, and is known to promote the expression of IL-10, IL-6 and VEGF, factors which are purported to assist in the induction of an immunoevasive TME (149). Therefore, combined assessment of both TIL-specific and tumor-derived molecules in surgical biopsies might uncover biomarkers which can predict patient outcomes or response to therapy with high accuracy. Finally, it is relevant to note that while scRNAseq and mass cytometry can unveil the diversity of the immune contexture in tumors in exquisite detail, these technologies offer no information on spatial configuration. However, a number of next generation, *in situ* profiling methods have recently been described which might offer improved capacity to detect immune cell subsets with prognostic and predictive potential for cancer.

## Next Generation Approaches for TIL Profiling

An overview of the published literature on TIL profiling shows that measuring TIL densities and phenotypes offers important mechanistic insight into tumor immunology (12, 23). Furthermore, TIL profiling has shown to offer prognostic value for patient outcomes as well as provide predictive information about successful response to ICB (12, 135, 138). However, the literature also reveals that there is a lack of consensus on methods for the identification, enumeration and scoring of TIL subsets, even in the context of well-studied tumor types such as melanoma (12, 30). Furthermore, in order to discover novel spatial relationships and phenotypic details, highly multiplexed approaches are required. Thus, this section will discuss two critical aspects of next-generation profiling approaches in cancer and their use in cutaneous melanoma. First, we will review a novel scoring algorithm for tumor infiltrating immune cells labeled “Immunoscore,” which has already been shown to have excellent prognostic potential and reproducibility in colorectal cancer (150, 151). Second, we will examine novel *in situ* immunophenotyping technologies which harbor significant promise for unveiling the complexity of the TME, particularly the immune contexture.

### Immunoscore

Currently, IHC-based detection of various TIL-specific markers (e.g., CD3, CD8, FOXP3) on serial sections of tumor tissue is one of the primary approaches for profiling the lymphocyte infiltrates in the TME (10, 12). As such, assessing lymphocyte densities as ratios such as CD8/FOXP3 or CD4/CD8 has been investigated and has shown to have prognostic potential in certain tumor types, but warrants further validation and systematic investigation (152, 153). However, a novel scoring algorithm for intratumoral lymphocytes termed the “Immunoscore” has been established as a potent prognostic tool in colorectal cancer (150, 154). Immunoscore ranges on a scale from I0-I4 and is based on digitally quantified densities of IHC-labeled CD3^+^ and CD8^+^ T cells, both in the tumor center (CT) and at the invasive margin (IM) (150, 154). Using a dedicated image analysis software (Immunoscore^®^ Analyzer, HalioDx, France), an operator defines specific regions (tumor, healthy tissue, necrosis etc.) and validates the CD3 and CD8 stains (154). An IM extending 360 μm into the healthy tissue and 360 μm into the tumor is automatically determined by the software (154). In stage I, II and III colorectal cancer, Immunoscore performed better as a prognostic factor for DFS, OS and DSS compared to the AJCC/IUCC (American Joint Committee on Cancer, International Union for Cancer Control) TNM staging system which examines the primary tumor (T), LN (N) involvement and metastases (M) (154). In a recent international, multi-center study from over 13 countries, colorectal cancer tissue samples from over 2,500 patients were analyzed by Immunoscore demonstrating high reproducibility and reliability of the assay across different centers (151). Furthermore patients with a high Immunoscore had a significantly reduced risk of disease recurrence at 5 years compared to patients with a low Immunoscore (151). Given that the Immunoscore assay qualifies most of the characteristics of an ideal biomarker (rapid, robust, reproducible, quantitative etc.), it has received regulatory approval for clinical use in colon cancer as an *in vitro* diagnostic and it has been advocated to include an immune component to TNM staging (TNM-I) in order to provide a more comprehensive prognostic assessment (154). Given the utility of Immunoscore in colon cancer, there have been efforts to translate it to additional tumor types such as melanoma, breast cancer and NSCLC (155). As such, the clinical validation of Immunoscore as a prognostic and predictive biomarker (for ICB) in these other tumor types are still underway while additional TIL markers such as CD20 and FOXP3 have also been included in evaluating the Immunoscore in melanoma (155). An additional aspect of adapting Immunoscore for prognostic use in advanced cancers, including melanoma is assessing its utility in biopsies from metastatic lesions where the IM is rarely available (154). However, Immunoscore has been adapted and tested for biopsies without IM and was shown to be prognostic as well in colorectal cancer, although this warrants further investigation (154). Ultimately, it is crucial to note that while CD8^+^ T cells are an essential component of tumor immunity, a number of additional immune cell types have shown prognostic associations in cancer such as FOXP3^+^ T_reg_, CD20^+^ B-TIL as well as myeloid cells such as M1 and M2 macrophages (10). Taken together, as Immunoscore has yet to be further studied for melanoma and a number of other tumor types, incorporation of additional immune markers into a more comprehensive Immunoscore might possess significantly enhanced prognostic potential for clinical outcomes or predictive utility for ICB.

### State-of-the-Art TIL Profiling

As novel imaging and molecular technologies emerge, the capacity to assess multiple markers simultaneously offers significant promise for discovering actionable and clinically relevant immune markers in cancer. Studies have shown that it is now possible to assess multiple RNA molecules at a single-cell resolution using single-molecule FISH (fluorescent *in situ* hybridization), and a recent report has demonstrated an iterative immunofluorescence (4i) based approach for multiplexed profiling of up to 40 proteins simultaneously (156, 157). However, these technologies are in their early phase of development and their application to tumor tissue immunophenotyping *in situ* has not been tested. In recent years, a series of detection methods involving cyclic immunofluorescence, nucleotide tagging or metal ion tagging as well as a number of novel image analysis softwares have been developed for simultaneous detection of multiple protein markers (as reviewed recently) (158). NanoString Technologies has also recently revealed the GeoMx^TM^ digital spatial profiling (DSP) technique, where mRNA or antibody probes are bound to photocleavable oligonucleotides (159). Using digital micromirror devices, ultraviolet light is guided to specified regions of interest in tissue labeled with fluorescent markers to visualize tumor (e.g., PanCK) and immune (e.g., CD45) regions (159). However, although this technique permits the spatially resolved simultaneous detection of large numbers of RNA or protein markers in FFPE tissue, it does not permit multiplex visualization of multiple markers on single cells (159). Given that the focus of this section is about technologies which can provide detailed immunophenotyping of TIL *in situ*, we will briefly discuss the nature of two major techniques which are currently in development for the simultaneous visualization of multiple protein markers in FFPE tissue (158).

Due to the fact that immunofluorescent labeling techniques are still used extensively in clinical cancer research, it is pertinent to briefly describe the methodologies currently in use for multiplexing using immunofluorescence. Currently, one technique for multiplexed immunofluorescence (mIF) in tumor tissue is based on sequential staining using tyramide coupled to a fluorophore, such as the Opal^TM^ system by PerkinElmer (160). Briefly, a single primary antibody is labeled using the Opal^TM^ HRP polymer and a single fluorophore followed by stripping of the bound antibodies using a special microwave (160). Using this approach, it is possible to multiplex up to 9 markers on the same cell, thereby yielding far more important on the TME compared to single or dual color IHC (160). Another recently described approach which demonstrates significant potential for multiparameter immunophenotyping of FFPE tissue tissue-based cyclic immunofluorescence (t-CyCIF), uses sequential rounds of immunofluorescence imaging in 4 channels to assess up to 60 markers (161). This process involves labeling and imaging with 3 antigens per cycle with a DNA marker to locate and register images across all cycles. This is followed by bleaching the fluorophores in the presence of white light before another cycle of immunolabeling. Finally all image tiles are stitched together and analyzed (161). The t-CyCIF approach has significant value for TIL profiling in human tumor biopsies. The repeated labeling and bleaching steps are labor-intensive and therefore the reproducibility of this approach across various research groups has not yet performed comprehensively. Nonetheless, t-CyCIF is a cutting-edge approach for multiplexed immunophenotyping and warrants further development.

Imaging mass cytometry (IMC) is an adaptation of mass cytometry (CyTOF), a technique which has utilizes metal isotope tags chelated to a polymers which are in turn conjugated to monoclonal antibodies, which are ionized and then measured on a single-cell basis using time of flight (TOF) mass spectrometry (158, 162). The stable metal isotopes are derived from the lanthanide series for a total of 37 unique metals, which can be used in combination with non-lanthanide metals (bismuth, gold, platinum) to create a panel of 40 markers which may be simultaneously assessed. IMC involves the labeling of immobilized tissues or cells on slides with mass cytometry antibodies which are then ablated using a pulsed laser and the resulting particles are transported to the mass cytometry via a stream of inert gas (162). The metal isotopes are all simultaneously detected and indexed for the specific location of the spot and an image is generated using the ion current for each mass tag to indicate the abundance of that tag (162). All of the detected markers are co-registered in computer generated images and advanced image analysis software is employed to segment and classify individual cells (162). Thus far, IMC has been applied to dissect the microenvironments of normal human and murine tissues as well as cell signaling pathways (162). Recent studies also reported the use of IMC on human FFPE tissues (LN, Hodgkin lymphoma and colon cancer), demonstrating its utility for providing multiparametric immune profiles of tumor tissue including microenvironmental features such as TLS in colon cancer, which were found to have high numbers of FOXP3^+^ T_reg_ (163). As IMC does not involve fluorescence, issues such as fluorescence spectral overlap and sample autofluorescence are obviated (158, 162). However, there are certain limitations to IMC, in particular, given that most mass cytometry antibodies are optimized for single cell suspension and not FFPE tissue and that image acquisition by laser is highly time intensive (1.5 mm^2^ in 2 h) (158, 162).

Co-detection by indexing (CODEX) is a multiplex fluorescence-based imaging approach which utilizes oligonucleotide conjugated antibodies (158, 164). Up to 50 antibodies can be assessed simultaneously and the sample is stained with the entire array of oligonucleotide-tagged antibodies (158). Each oligonucleotide is custom prepared with a 5’ overhang which allows for the incorporation of a fluorescent dye labeled nucleotide permitting the antibodies which are to be detected first having shorter overhangs than those which are set to be revealed later (158). A company which is commercializing CODEX technology, Akoya Biosciences^1^, has been established and offers an instrument which can be integrated into existing fluorescent microscopes for assay automation and image acquisition (165). While the technology offers several advantages such as obviating autofluorescence and simultaneously assessing multiple markers, the methodology is limited due to the sampling time and the fact that it has yet to be optimized for FFPE tissue (158). Nevertheless, data have been obtained for the application of CODEX in FFPE cancer tissues including specialized image analysis pipelines (165). Both IMC and CODEX have high potential for immunophenotyping of the TME; however, optimization of the methodologies as well as pipelines for the analysis and interpretation of the data obtained from these multiplex technologies require significant further investigation. Determining whether spatial configuration such as the distance between various immune and non-immune cell types, or the density of a specific TIL subset have association with patient clinical parameters such as disease stage or survival will be essential for revealing the translational value of these technologies (158).

## Concluding Remarks

A tumor’s capacity to evade immune control is now recognized as a canonical “hallmark” of cancer (166). In this review, we presented an overview of histopathological analysis of key lymphocyte subsets in cutaneous melanoma. Moreover, we have presented recent findings which offer tremendous potential in improving TIL profiling both for routine pathology and oncology research. A comprehensive classification of TIL in H&E stained melanoma tissue has been available since 1989 (23). Furthermore, multiple lymphocyte subsets, in particular, CD8^+^ T cells have shown both prognostic value for clinical outcomes, as well as predictive value for response to ICB (12, 30, 125, 138). Nevertheless, in the absence of large multi-center studies, standardized IHC protocols and automated enumeration methods, it will be difficult to validate the potential of various TIL subsets as prognostic and predictive biomarkers in cancer. Furthermore, studies have shown that tumor associated myeloid cells such as DC, macrophages, neutrophils and MDSC also play vital roles in modulating lymphocyte recruitment to the tumor (44, 167).

## Author Contributions

MS, FM, and HS conceptualized the study. MS oversaw the literature review was involved in all aspects of designing and writing the manuscript. FM performed the literature review. FM and HS wrote the manuscript. SS and RH provided immunopathology images and provided input on the discussion of various sections. All authors contributed to the article and approved the submitted version.

## Conflict of Interest

The authors declare that the research was conducted in the absence of any commercial or financial relationships that could be construed as a potential conflict of interest.

## Footnotes

1 https://www.akoyabio.com
